# Guidance of attention by statistical learning when multiple guidance mechanisms compete

**DOI:** 10.3758/s13414-026-03313-9

**Published:** 2026-08-02

**Authors:** Mark W. Becker, Morgan Dodd, Tristan Janisse

**Affiliations:** Department of Psychology, Michigan State University, Psychology Building, 316 Physics Dr., East Lansing, MI 48824 USA

**Keywords:** Statistical learning, Allocation of attention, Using multiple cues, Visual search

## Abstract

**Abstract:**

Previous research has shown that people are sensitive to statistical regularities and will implicitly learn to bias attention toward locations and features frequently associated with search targets. However, most prior work has involved a single biasing contingency. In Experiment 1, we investigate whether individuals can simultaneously learn and implement two distinct contingencies: one based on location and another based on color. The results suggest that both contingencies are learned implicitly and exert independent effects on attentional allocation. Experiments 2 and 3 examine whether shifting one feature to the volitional system via endogenous cues affects the implicit learning and implementation of contingencies for the other feature. The findings indicate that endogenous cues for one feature do not block implicit learning of contingencies in the other. However, the influence of implicit contingencies on attentional allocation depends on the validity of the volitional cues: they are effective when cues are neutral or valid, but not when invalid. This pattern suggests that the implicit and volitional systems operate hierarchically, with the volitional system taking precedence.

**Significance Statement:**

Efficient attentional guidance is key to effective visual search. While both volitional control and statistical learning can guide attention, little is known about how attention is influenced when multiple guidance mechanisms compete. Using a task with competing statistical regularities, one for target location and one for color, we show that both statistical learning biases independently influence attention. Shifting a feature to the volitional control system does not disrupt the statistical learning of the other, but invalid volitional cues eliminate statistical learning effects, suggesting a hierarchical relationship. These findings offer new insight into how multiple guidance systems shape attentional allocation.

**Open practices statement:**

Subject level data and SPSS analysis syntax available on the Open Science Framework at: https://osf.io/5vt74/overview. The experiments were not preregistered, but the data, and analysis syntax are publicly available at https://osf.io/5vt74/overview.

## Introduction

Given the vast amount of information in the visual environment, the brain must continuously prioritize a subset of information for additional processing while ignoring other information. The process by which this filtering occurs is thought to be attention, and one can only attend to a limited number of items at any given time. Given these capacity limitations, mechanisms that guide the allocation of attention toward task-relevant objects are critical for effective interaction with the environment. As a result, a substantial body of research has focused on understanding the mechanisms that control the allocation of attention to ensure that attentional resources are efficiently deployed.

For decades, attentional selection was thought to be determined through the interplay of two mechanisms – a bottom-up saliency-driven mechanism (Itti & Koch, 2000; Wang & Theeuwes, 2020) and a top-down goal-driven mechanism (Chen & Zelinsky, 2006; Folk et al., 1992). More recently, there has been growing appreciation for the role of a third mechanism, one that influences attentional allocation based on selection history (Anderson et al., 2021; Awh et al., 2012; Theeuwes, 2025). While often stated as a single third mechanism, these selection effects have come to encompass an array of factors that are likely distinct from one another. For instance, a review by Anderson and colleagues identifies seven different components of experience-driven attention that comprise at least three different types of learning: learning associations with valent outcomes (e.g., value-based capture), statistical learning (e.g., learning that frequent locations or features are associated with targets), and stimulus-response habit learning (e.g., attention to former targets).

Taken as a whole, this formulation of selection history can explain a number of phenomena not easily accounted for by a volitional top-down attentional mechanism, or a purely bottom-up saliency mechanism. It also accounts for findings that people are very sensitive to statistical regularities in the environment (Chun, 2000; Chun & Jiang, 1998; Theeuwes et al., 2022; Wang & Theeuwes, 2018) and that those regularities can help guide attention efficiently toward behaviorally relevant stimuli. Among the critical findings supporting the importance of these historical effects are results demonstrating that attention is biased toward features that have been associated with high reward (Anderson, 2013, 2016; Failing & Theeuwes, 2018), toward locations that are likely to contain targets (Chun & Jiang, 1998; Geng & Behrmann, 2005; Jiang, Swallow, Rosenbaum, et al., 2013a; Jiang et al., 2015), and toward features that are frequently associated with a search target (Conn et al., 2020; Schwark & Dolgov, 2013).

Importantly, much of this work suggests that these statistical regularities – or contingencies – are learned implicitly, without requiring explicit awareness of the contingencies (Chun & Jiang, 1998; Druker & Anderson, 2010; Geng & Behrmann, 2002; Jiang, 2018). This last point is important for establishing this historical mechanism as a separate mechanism from the top-down volitional mechanism – if historical context relied on explicit recognition of contingencies, one might argue that the explicit knowledge of the contingencies produced a volitional strategy of engaging the top-down system.

Despite the growing appreciation for the role that historical factors play in attentional allocation, some fundamental questions remain unresolved. One of these questions concerns whether multiple historical mechanisms can simultaneously bias attention.

What little research there is on the ability of multiple historical effects to simultaneously influence attention has focused on the question of whether value-based attentional capture effects are independent of the effects of statistical learning. Two studies associated a feature with a high reward, while simultaneously biasing attention away from a location with a frequent highly salient distractor, and found that the location-based mechanism based on statistical learning of the likely distractor location operated simultaneously and independently of the bias introduced by the reward manipulation (Kim & Anderson, 2021; Le Pelley et al., 2022). A third similar study extended this finding to demonstrate the independence of a valence-based mechanism and a statistical learning mechanism based on the frequent shape of a distractor (Ogden et al., 2023).

While these studies suggest that multiple types of selection effects might independently contribute to the allocation of attention, it is worth noting that in these studies the two factors influencing attention are from distinct learning mechanisms under the selection history umbrella – one from the valent learning mechanism and one from the statistical learning mechanism. Based on a variety of differences, including how context dependent they are (Anderson & Britton, 2019), how they influence anti-saccades (Kim & Anderson, 2019a), and their underlying brain mechanisms (Kim & Anderson, 2019b), it has been argued that the valence-based system is likely independent of statistical learning mechanisms associated with attention to former targets (see Anderson et al., 2021, for review). Thus, this leaves open the question of whether two factors from the same learning mechanism can simultaneously bias attention. A second limitation of these prior studies is that they all evaluated statistical learning by investigating the ability to avoid distraction by a salient distractor. The ability to use statistical learning to suppress a location may involve a different process to that of actively guiding attention toward a feature or location that is associated with the target.

Here we address these two issues by investigating the extent to which attention can be guided toward stimuli on the basis of two simultaneously presented statistical learning contingencies – one in which a given location is associated with a frequent target and the second in which a given color is frequently associated with the target. To foreshadow, in Experiment 1 we find evidence that both contingencies influence the allocation of attention and do so additively, suggesting independence. In Experiments 2 and 3 we turn to questions of whether moving one of the mechanisms into the volitional top-down system interferes with learning and implementation of the statistical learning associated with the other feature.

## Experiment 1

### Methods

#### Participants

G*Power was used to determine that 53 participants was the sample size necessary to achieve 95% power to find a moderate effect (partial eta squared =.06) in a repeated-measures ANOVA. Given that we were performing online recruitment, we anticipated ~20% attrition rate, so we ran a total of 66 participants through the experiment. The online platform Prolific (https://app.prolific.com) was used to recruit participants with the following screening criteria: Participants needed to be within the USA with English as their first language, had to participate on a laptop or desktop (no mobile devices), and had to be well-reviewed subjects on the platform (having a 95% approval rating or higher on pervious experiments they performed on Prolific). The experiment was approved by the Michigan State University Institutional Review Board (IRB) and adhered to additional Prolific guidelines. Each participant received financial compensation ($7.50) and the experiment took ~ 30 min.

#### Displays

**Fig. 1:**
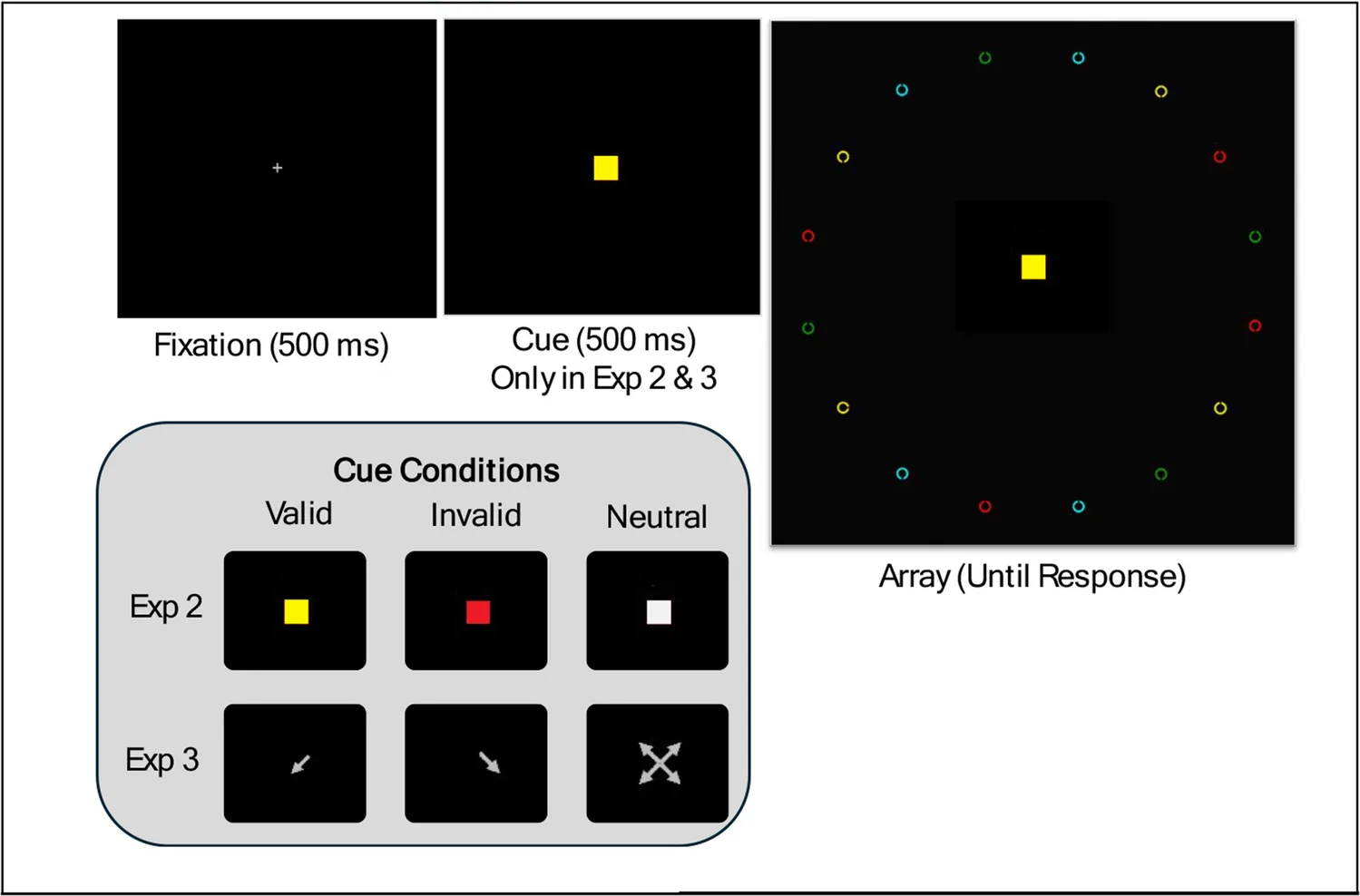
Schematic representation of the trials. The top row depicts an example of a validly cued trial in Experiment 2. Experiment 1 consisted of similar displays except the cue image was deleted and the fixation slide extended to 1,000 ms. The inset depicts the three types of explicit cues for Experiments 2 and 3 (given that the target in this example is yellow and in the lower left quadrant)

The experiment was programmed in E-Prime and run online via the E-prime Go platform. Trials began with the presentation of a small gray fixation cross in the center of a black screen (Fig. 1). After 1,000 ms this was replaced by a search array consisting of 16 Landolt Cs (just called Cs going forward) that remained on the screen until response. The Cs were presented equidistant around an imaginary clock face. The clock face was rotated 11.25 degrees from the vertical midline so that four Cs appeared in each quadrant of the display with no Cs along the horizontal or vertical meridian. Within each quadrant there was one C of each of four colors (Red, Green, Blue, Yellow) with the location of each color within the quadrant randomized. Fifteen Cs had breaks randomly appear on the top or bottom. The remaining C was the target and it had a break that randomly appeared on either the right or the left. Given that the experiment was run online, we do not know the size of the participant’s screen or viewing distance, so the size of the stimuli in visual angle is unknown and was variable across participants.

#### Contingencies

For each participant, one of the four quadrants was randomly chosen to be the dominant quadrant. During the training block the target appeared in that quadrant on 50% of the trials (72 trials), the target appeared in each of the other three quadrants in 16.67% of the trials (24 trials each). One color was also chosen at random to be the dominant target color for each participant. The target appeared in this color on 50% of the trials (72 trials) and in each of the other three colors on 16.67% of the trials (24 trials each). These two contingencies were applied independently, so the target in the dominant quadrant appeared 36 times in the dominant color, and 12 times in each of the three non-dominant colors. In each of the non-dominant quadrants, the target appeared 12 times in the dominant color and four times in each of the three non-dominant colors.

During the test block, these contingencies were removed. Thus, the target appeared equally often in each quadrant (20 times per quadrant), and within each quadrant the target appeared an equal number of times in each of the four colors (each color appeared as the target five times in each quadrant).

#### Procedure

The experiment began with written instructions informing subjects that their task was to search among a set of Cs with breaks on the top and bottom to find the one C with a break on the left or right and to hit the key corresponding to the side of the break (“M” key for right; “Z” key for left). They were told to respond as quickly as possible while maintaining accuracy.

After the instructions participants performed a short practice block of 16 trials to familiarize them with the task. During this block the target appeared equally often in each color and equally often in each quadrant of the screen. This practice block was followed by a 144-trial training block, which implemented location-based and color-based contingencies (see below). Finally, participants performed a test block of 80 trials in which the contingencies were removed. This final block allowed us to determine whether attentional biases created during training persist and control for the fact that there were unbalanced inter-trial priming effects in the training block.

After the last block of trials, participants completed a four-question questionnaire. One question asked whether the participant noticed that the target appeared more frequently in one quadrant of the screen than the others. The next questions presented an image with the four quadrants labeled with numbers from 1 to 4 and stated that the target appeared more often in one of the four quadrants, and asked participants to indicate the quadrant in which the target appeared most often by entering a number from 1 to 4. The next question asked whether the participant noticed that the target appeared more often in one color than the others, and this was followed by a four-alternative forced-choice question that asked the participant to indicate which color (Red, Green, Blue, or Yellow) the target appeared in most frequently by entering the corresponding letter for the color (R, G, B, Y).

### Results

#### Data filtering

For each experiment we eliminate the data from any participants whose overall accuracy was below 65%. This led to the elimination of data from 15 participants leaving a final sample size of 51. For reaction time (RT) analyses we eliminated extreme outliers > 16 s or < 300 ms and then calculated each subject’s mean RT and standard deviation for hit trials in the no-contingency block and eliminated trials with RTs > 3 standard deviations from the subject’s mean RT.

#### Accuracy

Accuracy rates were near ceiling, with accuracy rates > 96% for all conditions. Not surprisingly, there were no main effects or significant interactions for accuracy rates (Training Block: Color *F*(1, 50) = 0.02, *p* =.904, ${\eta}_{p}^{2} <.001$, Quadrant *F*(1, 50) = 0.11, *p* =.742, ${\eta}_{p}^{2} =.002$, Interaction *F*(1, 50) = 2.03, *p* =.161, ${\eta}_{p}^{2} =.039$; No Contingency Test Block: Color* F*(1, 50) = 0.46, *p* =.503, ${\eta}_{p}^{2}=.009$, Quadrant *F*(1, 50) = 0.03, *p* =.858, ${\eta}_{p}^{2} =.001$, Interaction *F*(1, 50) = 0.08, *p* =.777, ${\eta}_{p}^{2}=.002$.).

#### Reaction time

**Fig. 2:**
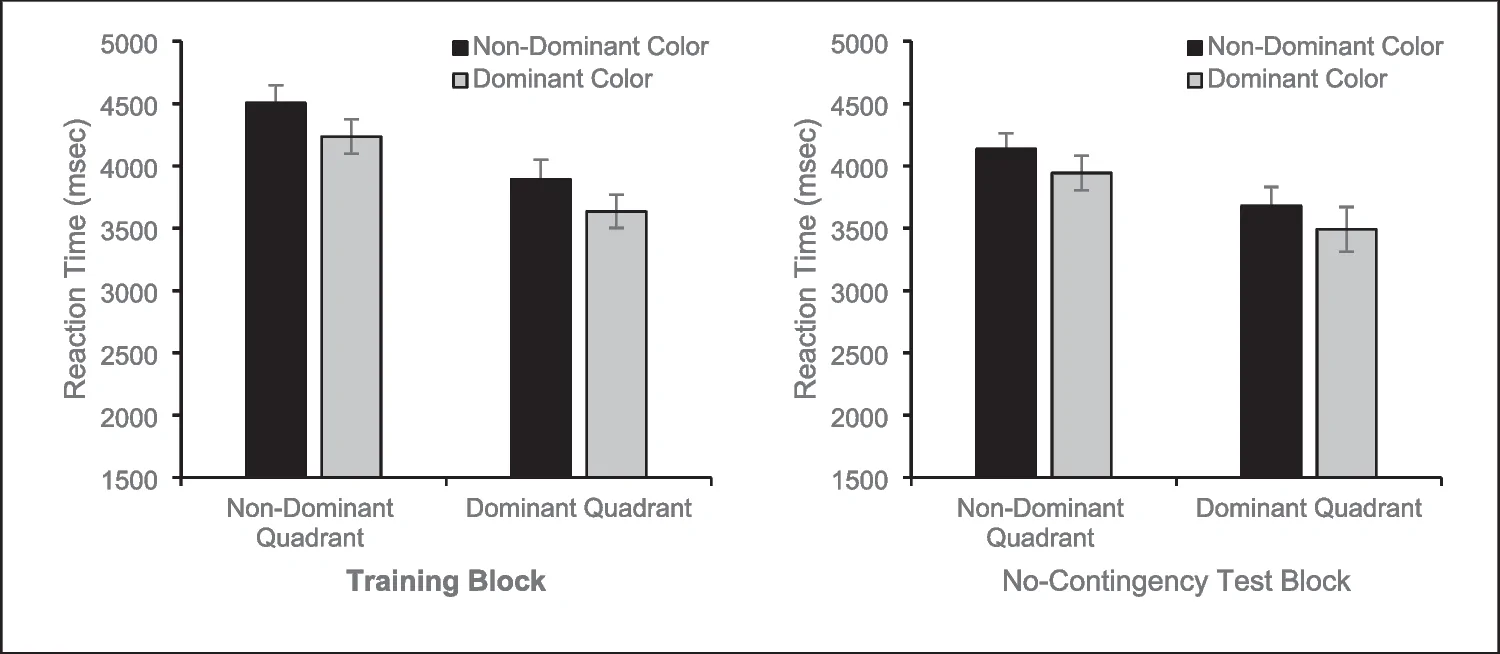
Mean reaction times as a function of whether the target appeared in the dominant color and/or location for the training block and the no-contingency block. Error bars indicate the standard error of the mean

We began with an omnibus 2 (Block: training/no contingency) x 2 (Color: Non-dominant/dominant) x 2 (Quadrant: Non-dominant/dominant) within-subjects ANOVA (see Fig. 2). There was a significant main effect of Block, *F*(1, 50) = 13.95, *p* <.001, ${\eta}_{p}^{2}=.22$, with faster RTs during the no-contingency test block (*M* = 3,812.6 ms, *SE* = 124.0) than the training block (M = 4,067.2 ms, *SE* = 128.0). This finding probably represents speeding up as one becomes more familiar with the task. More importantly, there was a main effect of color, *F*(1, 50) = 16.93, *p* <.001, ${\eta}_{p}^{2}=.25$, with faster RTs when the target appeared in the dominant color (*M* = 3,826.2 ms, *SE* = 123.8) than when it appeared in a non-dominant color (*M* = 4,053.6 ms, *SE* = 125.0). Similarly, there was a main effect of quadrant, *F*(1, 50) = 38.32, *p* <.001, ${\eta}_{p}^{2}=.43$, with faster RTs when the target appeared in the dominant (*M* = 3,674.7 ms, *SE* = 132.3) than in a non-dominant (*M* = 4205.0 ms, *SE* = 124.9) quadrant. None of the two-way or the three-way interactions approach significance, all *p* >.26, all ${\eta}_{p}^{2}<.026$. This pattern of results suggests that the color and location contingencies both influenced attention, did so independently, and the influence of each maintained during the no-contingency test block.

To further verify that the effect occurred in each block, we also ran follow-up 2 (Color) x 2 (Quadrant) ANOVAs for each block. Consistent with the above finding, there was a main effect of color and quadrant for each block [training: color *F*(1, 50) = 20.98, *p* <.001, ${\eta}_{p}^{2}=.30$, quadrant *F*(1, 50) = 40.46, *p* <.001, ${\eta}_{p}^{2}=.45$; no-contingency test: color *F*(1, 50) = 5.18, *p* =.03, ${\eta}_{p}^{2}=.09$, quadrant *F*(1, 50) = 13.99, *p* <.001, ${\eta}_{p}^{2}=.22$]. In neither block did the factors interact, both *F* < 1.

These data suggest that both factors can be learned implicitly and have independent and additive impacts on attention. Further, although the effects are somewhat diminished in the no-contingency block, the fact that they remain significant suggests that the effect is due to an attentional bias rather than inter-trial priming effects.

As a further test of additivity, we conducted Bayesian repeated-measures ANOVAs in JASP (v0.97) using default prior settings. These defaults specify multivariate Cauchy priors on fixed effects (*r* = 0.5), with all models including subject and random slopes for repeated-measures factors. Inclusion Bayes factors for the Color × Quadrant interaction were consistent with additivity, although evidence favoring exclusion of the interaction was only anecdotal (BF_incl_ =.88 for the training phase and.38 for the test phase).

Because inclusion Bayes factors average evidence across the full model space – including models that omit one or more main effects – we additionally performed a more targeted comparison that directly assessed whether the interaction improved model fit beyond the two main effects. Specifically, we compared a model including both main effects (Color + Quadrant) to a model additionally including their interaction. In the training phase, the data were approximately 4.5 times more likely under the additive model (BF = 0.22 for the interaction model relative to the additive model). A similar pattern was observed in the test phase (BF = 0.22), again indicating that the data were approximately 4.5 times more likely under the model without the interaction. Taken together, these analyses provide consistent, albeit somewhat weak, evidence for additivity, favoring an account in which the effects of Color and Quad combine without an interaction.

Finally, to investigate whether these effects are truly implicit, we ran a post hoc analysis which included data from only participants who reported no explicit awareness of the contingencies. There were 36 participants who failed to choose the dominant color during the four-alternative forced-choice question. An analysis including only these subjects found that there was still a main effect of color in the training block, *F*(1, 35) = 12.26, *p* =.001, ${\eta}_{p}^{2}=.26$. Similarly, there were 32 participants who failed to choose the dominant quadrant during the four-alternative forced-choice question. An analysis including only these subjects found that there was still a main effect of quadrant in the training block, *F*(1, 31) = 20.65, *p* <.001, ${\eta}_{p}^{2}=.40$. The fact that the effects maintain even among those who do not become explicitly aware of the contingencies, suggests that the contingencies are learned implicitly. We also performed these analyses with only the unaware participants for the no-contingency block. Among those who were unaware of the dominant quadrant, there was still a main effect of quadrant, *F*(1, 31) = 6.58, *p* =.02, ${\eta}_{p}^{2}=.18$. Among those who were unaware of the dominant color, the effect of color was marginally significant, *F*(1, 35) = 3.02, *p* =.09, ${\eta}_{p}^{2}=.08$. The fact that the quadrant effect extended into the no-contingency block and the color did so marginally, is fairly impressive, given that we would expect the continency effects to dissipate once the contingencies were removed, and the power of these analyses were reduced due to only including half our subjects. Thus, we take these relatively persistent effects as further evidence that the contingencies are learned implicitly.

### Discussion

The results from Experiment 1 show that when there are two separate contingencies, one for the target’s likely color and a second for the target’s likely location, both contingencies can be learned and both independently bias attention. Further, the fact that the effects persist, at least for a short duration, once the contingencies are removed eliminates a potential explanation based on inter-trial priming effects. Finally, the fact that the effects occurred even among subjects who appeared to be explicitly unaware of the contingencies suggests that these biases occur implicitly based on statistical regularities, rather than depending on explicit knowledge driving volitional attention. However, this last point raises the question of whether including a volitional top-down component would influence the learning and application of these implicit biases.

A number of researchers have investigated how engaging the volitional top-down attention system may influence the allocation of attention based on statistical learning; however, the results are contradictory. For instance, Wang and Theeuwes had people perform a search for a particular shape among heterogeneous distractors and had a salient distractor appear more often in one location. They found evidence that the salient distractor was more effectively suppressed when it appeared in its frequent location, suggesting that the statistical learning of location operated despite engaging the top-down volitional system via a search template. Similarly, Gao and Theeuwes had people keep a spatial location in working memory to engage the volitional system toward that location, while they performed a visual search in which either the target appeared frequently in one location (Experiment 1) or a salient distractor appeared frequently in one location (Experiment 2). In both cases, they found evidence that the spatial learning mechanism was not impeded by the engagement of the top-down system. By contrast, Dolci and colleagues (2023) paired an endogenous spatial cue with a high/low location manipulation and suggested that statistical learning is eliminated in the presence of a reliable endogenous cue. Jiang and colleagues (Jiang, Swallow, Rosenbaum, Herzig et al., 2013b) also reported that endogenous spatial cues eliminated the effect of statistical learning of a location, but a confound in their design raises questions about the reliability of that report (Jiang et al., 2022).

In short, the results are contradictory – with those that engage the top-down volitional system via a working memory task or search template seeming to show no evidence for interference with statistical learning, while those that engage the top-down mechanism through a more direct endogenous cue find that it interferes with statistical learning. However, it is worth noting that both studies that used an endogenous spatial cue paired it with a location based statistical learning manipulation, raising the possibility that statistical learning can occur in the presence of endogenous cues, but when both are location-based and contradict each other, preference is given to the volitional system.

Given that possibility, we used an endogenous cue for one feature (color or location) while the statical learning signal was for the opposite feature, to further investigate how engaging the volitional top-down system may influence a statistical learning mechanism’s influence on the allocation of attention.

In Experiment 2 we included an explicit cue about the likely color of the upcoming target and investigated whether doing so would block the learning and application of an implicit location-based contingency. Then in Experiment 3 we reversed this and made the likely location explicit to see how doing so would impact the implicit learning and application of a color-based contingency.

## Experiment 2

### Methods

#### Participants

Seventy-four participants were again recruited online via Prolific. The experiment was again approved by the Michigan State University IRB, and participants received identical compensation to that in Experiment 1, prorated to the duration of their participation. Of the 74 participants administered the experiment, nine did not complete the program and produce data, reducing the sample to *n* = 65.

#### Procedure

The method in Experiment 2 was similar to that in Experiment 1, except that the fixation screen was only presented for 500 ms and then an explicit color cue was presented for 500 ms before the array appeared. Once it onset, the cue maintained its location in the middle of the screen until the entire array was cleared following response.

The practice block was similar to Experiment 1, except that the trials include the explicit color cue. In the 16 practice trials the cue was valid 75% of the time (12 trials) and invalid and neutral 12.5% of the time (two trials each). The target appeared equally often in each of the four quadrants.

Similarly, in the training block the color cue was valid 75% of the time, invalid 12.5% of the time, and neutral 12.5% of the time. Within each cue condition, the target appeared equally often in each of the four colors – that is, color was endogenously cued but no longer associated with any contingencies that should bias attention toward a specific color. However, location had the same contingencies as in Experiment 1 – with the target appearing in a dominant quadrant on 50% of trials and in each of the other three quadrants on 16.67% of the trials. The location of the dominant color was randomized across participants. The location contingencies were counterbalanced across the three types of color cues, to make the two factors independent of each other. To accomplish this counterbalancing we had to increase the number of trials in the training block to 192. Doing so resulted in the following number of trials for each cell of the design. There were 36 validly cued trials for each target color, with 18 of those trials having the target appear in the dominant quadrant, and six trials with the target appearing in each of the other three quadrants. There were six invalidly cued trials for each target color, in three of these trials the target appeared in the dominant quadrant, and the target appeared in each of the other three quadrants once. Similarly, there were six neutrally cued trials for each target color, in three of these trials the target appeared in the dominant quadrant, and the target appeared in each of the other three quadrants once.

The test block was identical to the test block from Experiment 1, with the target appearing equally often in each color and quadrant. The only difference was that every trial in the test block began with a neutral cue.

After completing the trials, participants answered only the two questions probing their knowledge about the dominant quadrant.

### Results

#### Data filtering

We applied the same filtering criteria as for Experiment 1. Based on overall accuracy rates < 65% data from 27 participants were eliminated from further analysis, leaving a final sample size of 38. For RT analyses we eliminated extreme outliers > 16 s or < 300 ms and then calculated each subject’s mean RT and standard deviation for hit trials in the no-contingency block and eliminated trials with RTs > 3 standard deviations from the subject’s mean RT.

#### Accuracy

A 3 (Color Cue Validity: Invalid/Neutral/Valid) x 2 (Quadrant: dominant/non-dominant) within-subjects ANOVA on the training block found a main effect of Color Cue Validity, *F*(2, 74) = 9.75, *p* <.001, η^2^ₚ =.21, with higher accuracy for valid cues (*M* = 95.1%, *SE* = 1.5%) than neutral cues (*M* = 88.8%, *SE* = 2.6%), and invalid cues (*M* = 85.7%, *SE* = 3.0%). Neither the main effect of Quadrant nor the interaction approached significance, both *F* < 1, both η^2^ₚ <.004. In the no-contingency test block the color cue was always neutral, so the only comparison was a paired comparison of when the target appeared in the formally dominant quadrant to when it appeared in a non-dominant quadrant. This comparison failed to approach significance, *t*(37) =.74, *p* (one-tailed) =.23.

#### Reaction time

**Fig. 3:**
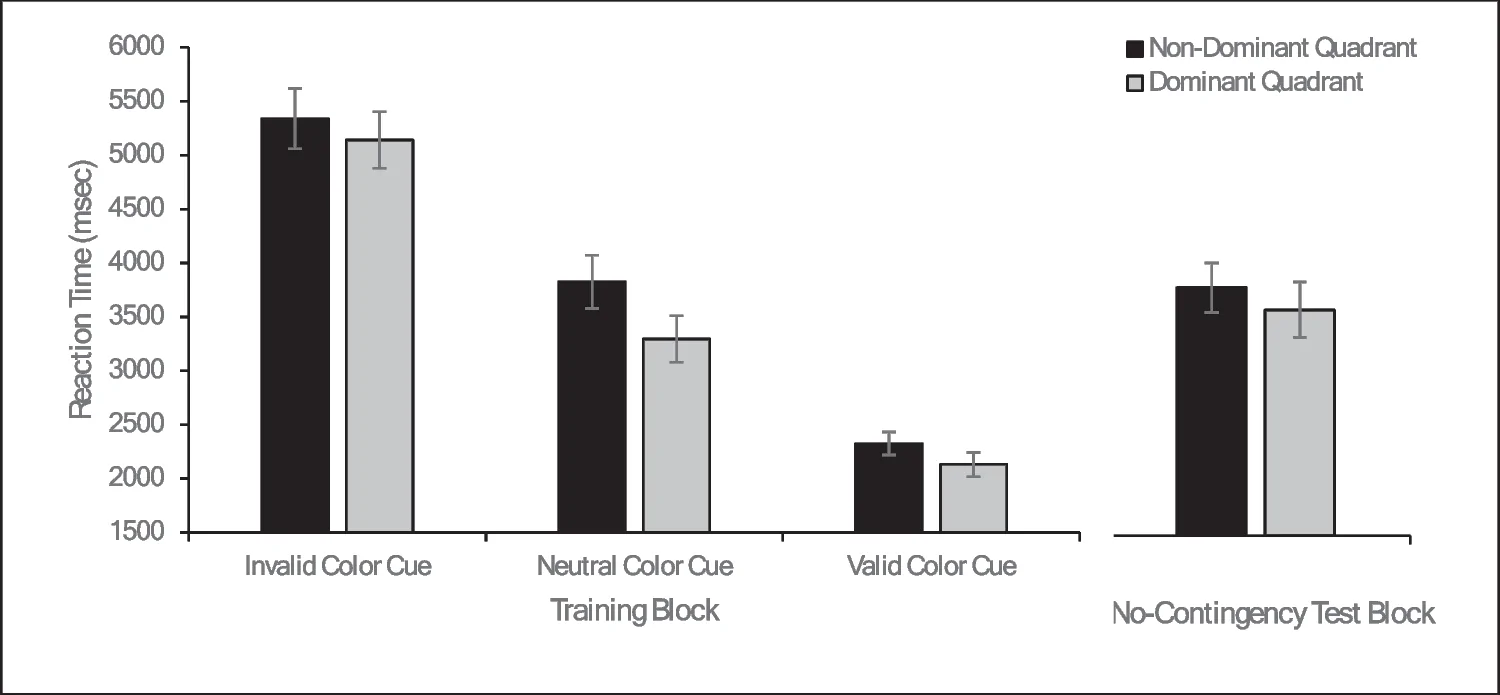
Mean reaction time is plotted as a function of cue validity and quadrant for the training blocks. On the right we also show the effect if quadrant in the No-Contingency Test Block, when the color cue is always neutral

A 3 (Color Cue Validity: Invalid/Neutral/Valid) x 2 (Quadrant: dominant/non-dominant) within subjects ANOVA (see Fig. 3) on the training block found a main effect of Color Cue Validity, F(2, 74) = 122.38, *p* <.001, ${\eta}_{p}^{2}=.77$, with faster RTs in the valid condition (*M* = 2228.2 ms, *SE* = 102.4 ms) than the neutral (*M* = 3.560.6 ms, *SE* = 319.6 ms) and invalid (*M* = 5,239.9 ms, *SE* = 258.1 ms) conditions. There was also a main effect of Quadrant, *F*(1, 37) = 13.85, *p* <.001, ${\eta}_{p}^{2}=.27$, with faster RTs when the target appeared in the dominant quadrant (*M* = 3,522.8 ms, *SE* = 167.1 ms) than the non-dominant quadrants (*M* = 3,829.7, *SE* = 185.0 ms). The two factors did not interact, *F*(2, 74) = 2.32, *p* =.11, ${\eta}_{p}^{2}=.06$. Planned pair-wise comparison showed that the effect of Quadrant was significant when the color cue was neutral, *t*(37) = 3.67, *p* (one-tailed) <.001, *d* =.60, and when the color cue was valid, *t*(37) = 2.60, *p* (one-tailed)=.007, *d* =.42. However, when the color cue was invalid, the effect of Quadrant was no longer significant, *t*(37) = 1.23, *p* (one-tailed) =.11, *d* =.20. There were 28 participants who failed to guess the dominant quadrant during the four-alternative forced-choice question. Using that response as a proxy for awareness of the location contingencies, we limited our analyses to these unaware subjects, and found that even among them there was an effect of quadrant in the training task, *F*(1, 27) = 9.63, *p* =.004, ${\eta}_{p}^{2}=.26$, with faster RTs when the target appeared in the dominant quadrant (*M* = 3,333.6 ms, *SE* = 179.1 ms) than other quadrants (*M* = 3,621.2 ms, *SE* = 208.8 ms), suggesting that the location-based effect is not due to explicit knowledge driving volitional attention. Consistent with the complete analysis, there was also a main effect of Cue Validity, *F*(2, 54) = 71.72, *p* <.001, ${\eta}_{p}^{2}=.73$, and the two factors did not interact, *F*(2, 54) = 1.41, *p* =.25, ${\eta}_{p}^{2}=.05$.

In the no-contingency test block, there was an effect of quadrant, *t*(37) =1.83, *p* (one-tailed) =.038, *d* =.30, with faster RTs when the target appeared in the previously dominant quadrant (*M* = 3,592.5 ms, *SE* = 257.2 ms) than the other quadrants (*M* = 3,799.0 ms, *SE* = 229.7 ms).

### Discussion

The pattern of results suggests that the statistical learning of the frequent target location was, for the most part, maintained even in the presence of an endogenous color cue. The dominant color produced faster RTs during the no-contingency test block, and for neutral and valid cues. The only time the bias toward the frequent location did not appear was when the endogenous cue was invalid – meaning that the color of the actual target did not match that of the endogenous cue. Clearly the fact that statistical learning was apparent in most conditions demonstrates that the presence of the endogenous cue did not disrupt the learning of the statistical regularities. However, the fact that statistical learning did not impact the allocation of attention when the explicit cue was invalid may have important implications for how engagement of the endogenous system may gate the ability of the statistical learning mechanism to influence the allocation of attention. Before further consideration of these potential implications, we first sought to determine how consistent they were. That is, whether a similar pattern of results would occur if the likely location of the target was made explicit through an endogenous cue while the color contingency was relegated to statistical learning.

## Experiment 3

### Methods

The methods for Experiment 3 were identical to Experiment 1 except here location was the explicitly cued factor, and the color contingencies were counter-balanced with the three types of explicit location cues. The location cues consisted of a central arrow that pointed to one of the four quadrants (valid and invalid conditions) or all four quadrants (neutral condition). Finally, the comprehension questions at the end assessed participants’ awareness of the color-based contingency rather than the location-based one.

#### Participants

For Experiment 3 we recruited 60 participants on prolific, with 58 of them completing the experiment and producing valid data. Similar to Experiments 1 and 2, the third experiment was approved by Michigan State University IRB and outlined an identical compensation platform.

### Results

#### Data filtering

We applied the same filtering criteria as for earlier experiments. Data from 29 participants were eliminated from further analysis due to overall accuracy rates below 65%. Again, this attrition rate was unexpectedly high, but given the large effect size we found in earlier experiments the sample should still be adequately powered.

#### Accuracy

Like Experiment 1, accuracy rates were near ceiling, with accuracy rates > 96% for all conditions. A 3 (Location Cue Validity: Invalid/Neutral/Valid) x 2 (Color: dominant/non-dominant) within-subjects ANOVA on the training block found no main effect of cue validity, *F*(2, 56) =.34, *p* =.72. There was also no main effect of color of the target, *F*(1, 28) =.54, *p* =.47, ${\eta}_{p}^{2}=.02$, nor a significant interaction, *F*(2, 56) =.14, *p* =.87, ${\eta}_{p}^{2}=.005$*.* In the no-contingency test block the location cue was always neutral, so the only comparison was a paired comparison of when the target appeared in the formally dominant color to when it appeared in a non-dominant color. This comparison was not significant, *t*(28) = 1.42, *p* (one-tailed) =.08, *d* =.26.

#### Reaction time

**Fig. 4:**
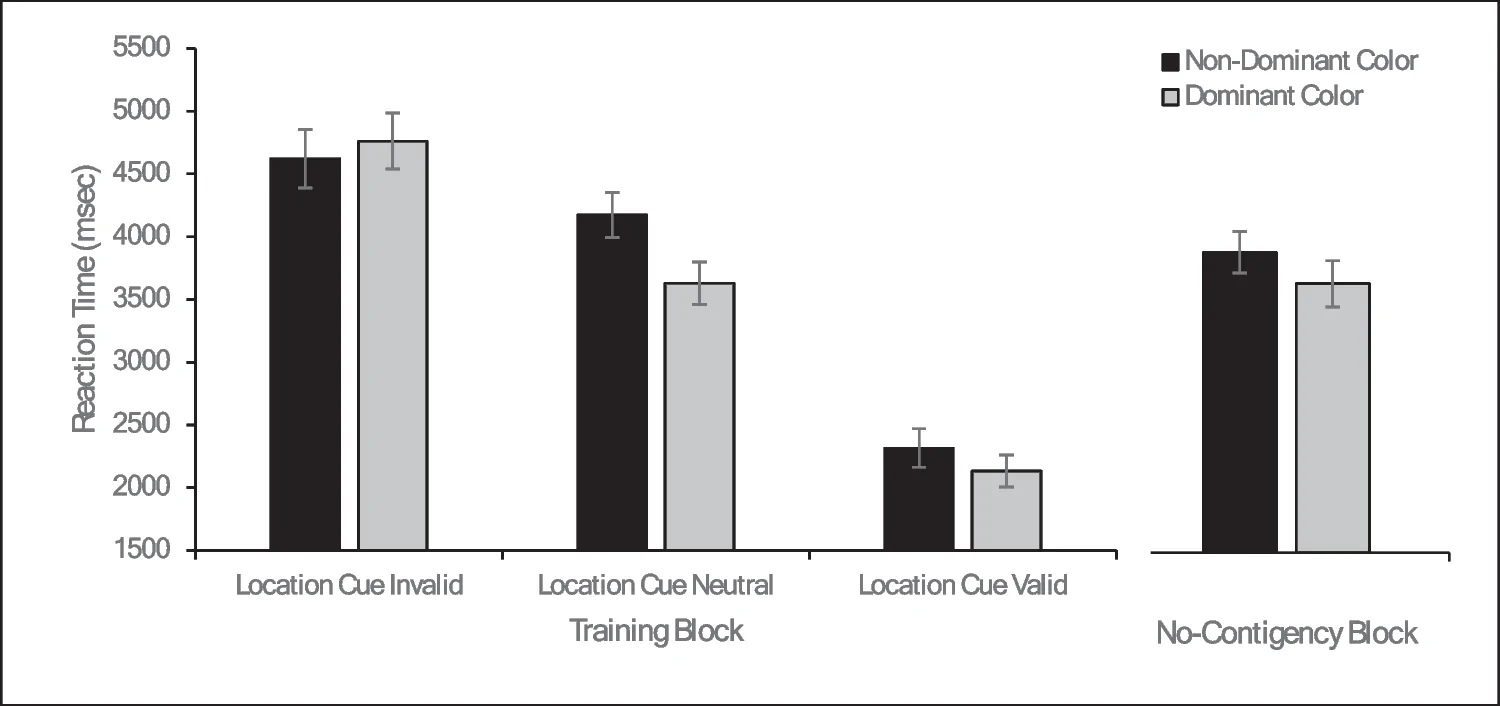
Mean reaction times are plotted as a function of location cue validity and target color in the training block. We also show the reaction time for the no-contingency block, in which all location cues were neutral, as a function of target color. Error bars are the standard error of the mean

A 3 (Location Cue Validity: Invalid/Neutral/Valid) x 2 (Color: dominant/non-dominant) within-subjects ANOVA (see Fig. 4) on the training block found a main effect of Location Cue Validity, *F*(2, 56) = 143.79, *p* <.001, ${\eta}_{p}^{2}=.84$, with faster RTs in the valid condition (*M* = 2,224.5 ms, *SE* = 139.1 ms) than the neutral (*M* = 3,901.6 ms, *SE* = 158.0 ms) and invalid (*M* = 4,691.6 ms, *SE* = 203.1 ms) conditions. There was also a main effect of Color, *F*(1, 28) = 4.44, *p* =.044, ${\eta}_{p}^{2}=.14$, with faster RTs when the target appeared in the dominant color (*M* = 3,507.9 ms, *SE* = 142.0 ms) than the non-dominant quadrants (*M* = 3,703.9, *SE* = 162.7 ms). The two factors also interacted, *F*(2, 56) = 5.61, *p* =.006, ${\eta}_{p}^{2}=.17$. Planned pairwise comparisons show that the source of the interaction was that targets in the dominant color were found more quickly when the location cue was valid, *t*(28) = 3.34, *p* (one-tailed) =.001, *d* =.62, or neutral, *t*(28) = 3.69, *p* (one-tailed) <.001, *d* =.69. However, having the target appear in the dominant color was not helpful when the location cue was invalid, *t*(28) =.67, *p* (one-tailed) =.25, *d* =.13.

There were 18 participants who failed to correctly identify the dominant color in the four-alternative forced-choice question. Restricting the analysis to only those 18 participants who were unaware of the dominant color still found a main effect of Color, *F*(1, 17) = 4.49, *p* = 049, ${\eta}_{p}^{2}=.21$. Consistent with the overall analysis there was also a main effect of location cue validity, *F*(2, 34) = 101.76, *p* <.001, ${\eta}_{p}^{2}=.86$. While the overall analysis did not produce a significant interaction, *F*(2, 34) = 1.916, *p* =.16, ${\eta}_{p}^{2}=.10$, the pairwise comparisons mimic the overall analysis, with the target being found more quickly when it appeared in the dominant color when the location cue was valid, *t*(17) = 2.24, *p* (one-tailed =.02, *d* =.53, or neutral, *t*(17) = 3.12, *p* (one-tailed) =.003, *d* =.74, but not when the location cue was invalid, *t*(17) =.095, *p* (one-tailed) =.46, *d* =.02.

In the no-contingency test block, there was an effect of color, *t*(28) = 2.54, *p* (one-tailed) =.009, *d* =.47, with faster RTs when the target appeared in the previously dominant color (*M* = 3,644.4.5 ms, *SE* = 183.9 ms) than in the other colors (*M* = 3,893.3 ms, *SE* = 166.1 ms).

## General discussion

Experiment 1 clearly established that statistical learning for two distinct factors (color and location) can be acquired simultaneously, and exert independent influences on the allocation of attention. This finding expands on prior work showing that two historical effects that involve separate systems – a reward system and a statistical learning system – can exert independent effects on attention (Kim & Anderson, 2021; Le Pelley et al., 2022), to the finding that two forms of statistical learning can also exert independent influences on attention. In addition, those experiments used relatively rapid or easy searches in which RTs took less than a second. By contrast, our experiment used a slow and demanding search task that likely required overt eye-movements and produced mean RTs greater than 3 s.^1^ As such, our results align nicely with a recent paper by Anderson (2026) that demonstrated additive effects of color-based and location-based statistical learning in a difficult T among L search task with set sizes as high as 50 items. Critically, their set-size manipulation allowed them to demonstrate that statistical learning led to a decrease in search slopes, suggesting that the biases influence search guidance. Together, our results and Anderson’s (2026) findings suggest that statistical learning is a robust mechanism that can exert its influence even under conditions requiring serial effortful search.

Our Experiments 2 and 3 investigated how volitional, top-down guidance created by an endogenous cue in one dimension (color or location) would influence the learning and application of statistical learning in the opposite dimension. In both experiments the use of an endogenous cue did not eliminate statistical learning, and in both, statistical learning influenced the allocation of attention in almost all conditions. Most telling, statistical learning influenced attention in the follow-up test blocks, when the contingencies were removed, suggesting that these effects are somewhat persistent and cannot be explained solely on the basis of intertrial priming. Taken as a whole, the results from across the experiments suggest that statistical learning is a very robust mechanism. It has impacts on slow searches that likely require overt shifts of attention and can bias attention even in the presence of a highly informative explicit cue.

Interestingly, in both Experiment 1 and Experiment 2, the one condition that did not show evidence of attentional guidance by statistical learning was the condition in which the endogenous cue was invalid. To understand this selective failure, we appeal to Wolfe’s Guided Search model (Wolfe, 1994, 2021). Under that model, multiple independent sources of search guidance, each with their own attentional weights, can impact the level of activation within a priority map. As applied to our Experiments 2 and 3, the relatively large impact of the explicit cue suggests that its input to the priority map has a relatively high weight, while the weight of the statistical learning component would be somewhat weaker. Still, the two factors should be additive, so that the activation in the priority map should be highest for the item that is consistent with both factors, followed closely by the other explicitly cued items. Items that were not cued by the explicit cue would not benefit from the large activation boost conferred by the cue, but un-cued items that were consistent with statistical learning would still benefit from the modest increase in activation it provides. This pattern of activations would produce the independent effects we find in the valid and neutral cueing condition. However, the selective failure of statistical learning to impact attention in the invalid trials suggests that the activation pattern in the priority map is altered for these trials.

While Guided Search as currently stipulated sets attentional weights at the beginning of a block (Wolfe, 2007), Wolfe acknowledges that a more sophisticated model would provide for updating of these weights, if for nothing else to allow for things like priming to influence selection. We can envision two methods of updating weights that could account for the modified activation pattern for invalid cue trials. One explanation posits that the repeated failures to find the target among the explicitly cued set causes a reconfiguration or resetting of the attentional weights impacting the priority map. Under this view, if search guidance is proving ineffective, the weights are abandoned. A second possible explanation posits that the attentional weights remain relatively stable, but the functional influence of guidance signals diminishes as search progresses. This assumption is consistent with findings that guidance diminishes over time (Becker et al., 2026; Motter & Belky, 1998), and the fact that as search progresses items with a lower priority signal are more likely to be selected (Zelinsky, 2008; Zelinsky et al., 2013). This reduction of weights with search time could be due to decay or because of the buildup of factors like inhibition of return (Klein et al., 2023). Despite different mechanisms being responsible for the change in the priority map, under either scenario, the initial benefit conferred by the statistical learning does not maintain past the selection of the explicitly cued items, thereby eliminating the impact of statistical learning in invalid trials.

Our data cannot distinguish between these two Guided Search–compatible mechanisms we propose.^2^ To do so will require methods that more directly track, perhaps via eye movements, the temporal dynamics of guidance during search.

Even so, either of these explanations may be able to account for the behavioral results of Dolci and colleagues (2023), who paired an endogenous spatial cue with a high-probability location contingency. In their study they only had valid and neutral endogenous cues. Like us, they found faster target detection times at a highly likely location with a neutral endogenous cue. Unlike us, they failed to find an effect of statistical learning with a valid cue. However, their valid cues indicated a single item, while ours indicated a set of four items. Based on our explanations above, their valid cue would have conferred a large advantage to the single cued item, leaving no room for a benefit from the statistical learning mechanism. By contrast, our statistical learning mechanism could still have an influence by biasing attention within the set of four endogenously cued items.

The inverse argument might explain why Vilanova- Goldstein and colleagues (2022) found that a fairly weak attentional bias based on agency persisted and was additive with an invalid endogenous cue. Again, they had only one item in the display that matched the endogenous cue, and thus the strong endogenous cue would only dominate the first attentional selection. This single attentional deployment may not have taken enough time to allow the weaker agency bias to dissipate or might not be enough evidence of search failure to cause a reset of search weights. In either case, these two experiments highlight that one might need cues that indicate multiple items in order to have the sensitivity required to see the effects we report here.^3^

Taken as a whole, our results suggest that statistical learning may be a powerful influence on the allocation of attention – it appears that at least two statistical contingencies can be learned simultaneously and produce independent influences on attention. These contingencies can still exert their influence when a powerful explicit cue engages the volitional system. When an explicit cue indicates multiple possible targets, statistical learning can bias the order of selection among those items. However, when the explicit cue is invalid, the bias conferred by statistical learning loses its ability to confer an advantage to un-cued items. Whether this failure results due to guidance weakening as search progresses, or due to a reconfiguration of attentional weights when the explicit cue fails, remains to be determined.

The finding that statistical learning’s impact seems constrained to items consistent with an explicit cue raises an additional question about whether statistical contingencies could be learned if they only exist during invalid trials, and therefore outside the items consistent with the explicit cue. For instance, if each color was equally likely to be the target when an endogenous spatial cue was valid, but one color was far more likely to be the target on invalid trials, would people still learn that contingency? Alternatively, would they learn to attend preferentially to that color only on invalid trials?

There are also remaining questions about how many statistical regularities can simultaneously be learned and influence attention. Here we demonstrated that at least two can provide independent influences on attention, but questions remain about how many contingencies could simultaneously drive attention and what the limiting factors would be. For instance, here the two statistical learning mechanisms tap into different features – location and color, but could one train multiple contingencies within a given feature? Research that varied the percentage of time a target appeared at a given location from 30% to 90% found a graded benefit of attention (Zhang et al., 2022), suggesting that the system is sensitive to specific probabilities; however, those manipulations were done with separate groups of participants rather than training with multiple locations within a trial with different priorities.

Finally, it is worth noting that in all our experiments the effects did not appear to depend on the explicit recognition of the highly likely locations or colors. When our analyses are constrained to only those who failed to correctly identify the high priority location or color during a four-alternative forced-choice question, the effects generally maintain. This is consistent with much of the work on selection history effects that suggest that contingencies can be learned implicitly and support the view that these mechanisms are distinct from the volitional, top-down system.

## Data Availability

SPSS data files of the processed subject level data for all experiments is provided on the Open Science Framework (OSF) at: https://osf.io/5vt74/overview

## Footnotes

1 The fact that our task likely required fixating individual stimuli to identify the target raises the possibility that the statistical learning here is impacting procedural learning, influencing the pattern of eye-movement deployments. However, without direct eye-movement data this is speculative.

2 We note that Vilanova-Goldstein et al. (2022) also found that the inclusion of a both a valid and an invalid color singleton disrupted the application of an agency bias, which might suggest that the color singleton also precipitates a reset of attentional weights.

3 Anderson’s (2026) findings that both color and location-based statistical learning reduced search slope additively and resulted in linear set-size functions with set-sizes ranging from 5 to 50 might argue against the model which rests on the assumption that guidance diminishes as search progresses. If guidance diminished one might have expected the set-size functions to become non-linear for larger set sizes that required multiple attentional deployments. However, the reaction times for that task were still fairly rapid (< 2,000 ms for the slowest search with set size 50), so a model based on diminishing guidance might still hold, if the decay is fairly slow.

## Code availability

SPSS syntax for all the comparisons in the article are available at https://osf.io/5vt74/overview
